# Selecting a PRO-CTCAE-based subset for patient-reported symptom monitoring in prostate cancer patients: a modified Delphi procedure

**DOI:** 10.1016/j.esmoop.2022.100775

**Published:** 2023-01-16

**Authors:** E. Feldman, F.J. Pos, R.J. Smeenk, H. van der Poel, P. van Leeuwen, J.M. de Feijter, M. Hulshof, T. Budiharto, R. Hermens, K.M. de Ligt, I. Walraven

**Affiliations:** 1 Department for Health Evidence, Radboud University Medical Center, Nijmegen; 2 Department of Radiation Oncology, The Netherlands Cancer Institute, Antoni van Leeuwenhoek, Amsterdam; 3 Department of Radiation Oncology, Radboud University Medical Center, Nijmegen; 4 Department of Urology, The Netherlands Cancer Institute, Antoni van Leeuwenhoek, Amsterdam; 5 Department of Urology, Amsterdam University Medical Centers, Amsterdam; 6 Department of Internal Medicine, The Netherlands Cancer Institute, Antoni van Leeuwenhoek, Amsterdam; 7 Department of Radiation Oncology, Academical Medical Center, University of Amsterdam, Amsterdam; 8 Department of Radiation Oncology, Catharina Hospital, Eindhoven; 9 Scientific Institute for Quality in Healthcare, Radboud Institute for Health Sciences, Radboud University Medical Center, Radboud University Nijmegen, Nijmegen; 10 Department of Psychosocial Research and Epidemiology, The Netherlands Cancer Institute, Antoni van Leeuwenhoek, Amsterdam, the Netherlands

**Keywords:** prostate cancer, adverse effects, patient-reported outcomes

## Abstract

**Background:**

Clinician-based reporting of adverse events leads to underreporting and underestimation of the impact of adverse events on prostate cancer patients. Therefore, interest has grown in capturing adverse events directly from patients using the Patient-Reported Outcomes (PROs) version of the Common Terminology Criteria for Adverse Events (CTCAE). We aimed to develop a standardized PRO-CTCAE subset tailored to adverse event monitoring in prostate cancer patients.

**Materials and methods:**

We used a mixed-method approach based on the ‘phase I guideline for developing questionnaire modules’ by the European Organization for Research and Treatment of Cancer (EORTC) Quality of Life group, including a literature review, and interviews with patients (*n* = 30) and health care providers (HCPs, *n* = 16). A modified Delphi procedure was carried out to reach consensus on the final subset selected from the complete PRO-CTCAE item library.

**Results:**

Fourteen multidisciplinary HCPs and 12 patients participated in the Delphi rounds. Ninety percent agreed on the final subset, consisting of: ‘ability to achieve and maintain erection’, ‘decreased libido’, ‘inability to reach orgasm’, ‘urinary frequency’, ‘urinary urgency’, ‘urinary incontinence’, ‘painful urination’, ‘fecal incontinence’, ‘fatigue’, ‘hot flashes’, ‘feeling discouraged’, ‘sadness’, and ‘concentration’*.* From 16 articles identified in the literature review, the following adverse events for which no PRO-CTCAE items are available, were included to the recommendation section: ‘nocturia’, ‘blood and/or mucus in stool’, ‘hemorrhoids’, ‘hematuria’, ‘cystitis’, ‘neuropathy’, and ‘proctitis’*.*

**Conclusions:**

The obtained PRO-CTCAE-subset can be used for multidisciplinary adverse event monitoring in prostate cancer care. The described method may guide development of future PRO-CTCAE subsets.

## Highlights

• Patient-reported adverse event monitoring in cancer patients could improve overall survival and health-related quality of life.
• We are the first to identify a PRO-CTCAE subset for adverse event monitoring during and after prostate cancer treatment.
• The PRO-CTCAE prostate cancer subset could facilitate remote monitoring during and after cancer treatment.
• The current study may serve as a guide for future development of tumor-specific PRO-CTCAE-based subsets.

## Introduction

Prostate cancer is the second most common malignancy in men worldwide, with ∼1.4 million new registered cases in 2020.^1^ It is estimated that the incidence of prostate cancer in Europe increases with ∼30% from 2015 to 2040.^2^ The incidence is associated with increasing age, with the highest incidence in men >65 years of age.^2^ Most patients are nowadays diagnosed in a curable stage, likely caused by increased awareness, and diagnostic and treatment improvements over the last decades.^3^ Five-year survival rates in European countries are currently 83%-88%.^2^^,^^4^

Prostate cancer treatment can have a significantly negative and potentially remaining effect on patients’ health-related quality of life (HRQoL). For prostatectomy, negative effects on urinary continence and erectile functioning are still reported after 5 years of follow-up. Radiotherapy is associated with nocturia and worse bowel function.^5^^,^^6^ Optimal strategies to monitor and treat adverse events of treatment are warranted to overcome declined HRQoL.

Currently, clinicians report adverse events using the Common Terminology Criteria for Adverse Events (CTCAE). However, this clinician-based approach is criticized: clinicians structurally underreport and underestimate (the severity of) adverse events.^7^, ^8^, ^9^ Furthermore, agreement between clinicians when reporting adverse events was found to be moderate, with intraclass correlation coefficients ranging from 0.46 to 0.71.^10^

To overcome the gap between what clinicians report and what patients experience, interest grows in capturing adverse events directly from patients using Patient-Reported Outcomes (PROs), which may enhance its precision and comprehensiveness. Recently, Basch et al. (2016) demonstrated that integrating PRO monitoring into routine care is associated with clinical benefits, including decreased emergency room visits, improved overall survival, and less hospitalization compared to clinician-reported adverse event monitoring.^11^ Subsequently, the National Cancer Institute (NCI) developed a PRO version of the CTCAE for use in clinical care and research settings. Their item library consists of 124 items evaluating the frequency, severity, and/or activity interference of 78 symptomatic adverse events.^12^

Implementing the full PRO-CTCAE library in daily clinical practice for adverse event monitoring, however, is demanding and inappropriate for every tumor type or treatment. Selecting adverse events relevant for cancer types and treatments from this library is critically important to measure without bias:^13^ not all adverse events are relevant for each study population, intervention, context, objective, and setting. Therefore, the purpose of this study was to develop a standardized PRO-CTCAE item subset for unambiguously monitoring adverse events in prostate cancer patients.

## Materials and methods

**Figure 1:**
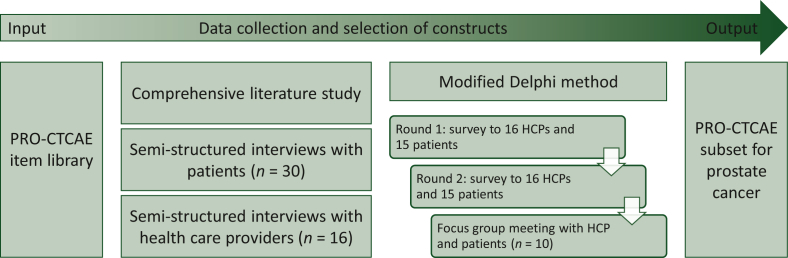
**Overview of the mixed-method approach for item identification and selection.** CTCAE, Common Terminology Criteria for Adverse Events; HCP, health care provider; PRO, Patient-Reported Outcome.

To develop a standardized PRO-CTCAE item subset, we used a mixed-method approach based on the ‘phase I guideline for developing questionnaire modules’ by the European Organization for Research and Treatment of Cancer (EORTC) Quality of Life group.^14^ This guideline was originally developed to select relevant items for inclusion in modules specific to tumor site, treatment modality, or HRQoL dimension. Our approach included a comprehensive literature study, and semi-structured interviews with patients (*n* = 30) and health care providers (HCPs, *n* = 16) to identify PRO-CTCAE items most relevant for prostate cancer patients. Subsequently, we used a modified Delphi procedure to reach consensus on the final selection of items (Figure 1). Additionally, analyses were executed stratified by treatment modality to determine whether there were items that cover treatment-specific adverse events. The Consolidated criteria for reporting qualitative research (COREQ) checklist was used for reporting about the interviews and focus groups.

The Netherlands Cancer Institute institutional review board (IRB) declared that formal approval from an ethics committee was not required, as the Dutch Medical Research (Human Subjects) Act did not apply for this study, and approved local execution of the study (IRBd19-334). All study participants gave informed consent for participating in the study.

### PRO-CTCAE item library

The input was the completely validated PRO-CTCAE item library. From the 78 PRO-CTCAE items, 74 items were applicable to men; four items applicable to women were excluded in further analyses (i.e. ‘irregular periods/vaginal bleeding’; ‘missed expected menstrual period’; ‘vaginal discharge’; ‘vaginal dryness’).

### Comprehensive literature study

A literature search was conducted to identify the most important adverse events and potentially missing PRO-CTCAE items relevant for prostate cancer. The following search terms were used in PubMed: ‘prostate cancer’ OR ‘prostatic neoplasms’ OR ‘prostatic adenocarcinoma’ AND ‘toxicities’ OR ‘adverse effects’ OR ‘side effects’ AND ‘radiotherapy’ OR ‘hormonal therapy’ OR ‘surgery’ OR ‘and chemotherapy’. See Supplementary Table S1, available at https://doi.org/10.1016/j.esmoop.2022.100775, for the full search strategy. Records were selected if they met the following inclusion criteria: studies that described adverse events, in patients treated for prostate cancer, measured through patient-reported outcome measures (PROMs) and/or other questionnaires. Prevalent and incident adverse events were retrieved from the final selected studies.

### Patient interviews

Prostate cancer patients who received curative treatment (*n* = 20) and palliative care (defined as patients with metastasized disease and/or not treated with curative intent; *n* = 10) between September 2020 and January 2021, recruited from community (*n* = 2) and academic hospitals (*n* = 2) in the Netherlands, were invited to participate in semi-structured interviews. The number of included patients was dependent on data saturation (the point where no new information emerged from the interviews). Purposive sampling was used to select a broad range of patients in terms of age, treatment type, and disease state. Inclusion criteria were: ≥18 years of age, currently undergoing primary prostate cancer treatment or ≤6 months after completing treatment, ability to provide informed consent, and ability to complete the questionnaire in Dutch or English; the latter two were based on the judgment of their treating physician or nurse. Interviews were systematically documented and recorded on audiotape by the interviewer.

Based on the EORTC guideline for developing questionnaire modules,^14^ we developed a scripted interview guide consisting of the following steps. Firstly, patients were asked to describe their symptoms. Subsequently, they completed the full PRO-CTCAE item library; the interviewer then asked what symptoms meant to them, the extent to which they had experienced these, and if they had experienced any other symptoms. Next, they were asked to score the relevance of each item on a four-point scale (1 = not relevant, 4 = highly relevant; items with a mean score ≥ 2 were considered relevant), and to comment on the item description wordings. Also, they indicated whether there were important adverse events missing in the PRO-CTCAE list.

### HCP interviews

HCPs treating prostate cancer patients were invited to participate in a semi-structured interview. The HCPs were equally distributed as specialists working in two community and two academic hospitals in the Netherlands, consisting of radiation oncologists, urologists, medical oncologists, and nurse practitioners.

A scripted interview guideline was developed following the EORTC guideline for developing questionnaire modules.^14^ Firstly, HCPs were asked to score the relevance of each PRO-CTCAE item on a four-point scale (1 = not relevant, 4 = highly relevant; items with a mean score ≥ 2 were considered relevant), and to comment on the item description wordings and to explain their score. Subsequently, they were asked to add items when they felt these were missing.

### The modified Delphi method

All medical specialists and patients who participated in the semi-structured interviews received an invitation to participate in the modified Delphi procedure. This is a method aimed at reaching consensus in a structured way, based on experts’ opinions on a specific subject. Information and feedback is gathered through multiple questionnaire rounds, which are completed anonymously to avoid bias that may result from desire to group conformity. The method is targeted at making best use of already available information.^15^ We followed the recommendations of the RAND modified Delphi method,^16^, ^17^, ^18^, ^19^ set up in three rounds. The first two rounds consisted of web-based questionnaires through an online survey program (Castor Electronic Data Capture). The results from each previous round were fed back anonymously in the introduction of the next round. The third round consisted of an expert and patient focus group meeting evaluating the results from the previous rounds. Lastly, all results and conclusions from the last round were sent to all participants for final consensus. The threshold for consensus was set at ≥75% agreement for each round. Items were excluded for the next round if <25% of both HCPs and patients considered an item to be relevant. If no consensus was reached, the item was transferred to the next round. The Delphi rounds are described in more detail in the following sections.

### Expert and patient recruitment

All medical specialists and patients who participated in the semi-structured interviews received an invitation to participate in the Delphi survey rounds. Of those, 14 HCPs (88%) and 12 patients (40%) agreed to participate.

### First Delphi round

The first round aimed to determine the relevance of the PRO-CTCAE items suggested in the interviews and retrieved from the literature. Questions were formulated uniformly and in the same order for HCPs and patients (from high to low relevance as scored during the interviews). For example: ‘Do you think that the adverse event “urinary frequency” is relevant and should be added in the final subset?’. Participants were asked to score the relevance of each item on a four-point scale (1 = not relevant, 4 = highly relevant; items with a mean score ≥ 2 were considered relevant). The consensus for items was calculated and summarized.

### Second Delphi round

The second round started with the summarized overview from the first round, administered as follows: (i) if ≥75% of patients but not HCPs scored an item as ‘relevant’, the item was only asked in the second round of the HCP survey, and vice versa; (ii) if an item scored between 25% and 75% in both expert groups, the item was asked in both HCPs and patients surveys. Participants were again asked to score the relevance of each item on a four-point scale (1 = not relevant, 4 = highly relevant; items with a mean score ≥ 2 were considered relevant).

### Third Delphi round

In the third Delphi round, we aimed to reach consensus for the final items and to evaluate the included and excluded PRO-CTCAE items from the previous rounds and to give recommendation for emerged adverse events that are currently not included within the PRO-CTCAE item list. In a focus group meeting including both patient representatives as well as a multidisciplinary HCP group, moderated by one of the coordinating investigators, the expert panel strived to create one subset for patients treated with curative or palliative intent.

### Finalization of the outcome subset

The final items list was presented to all Delphi participants by e-mail for formal agreement. Additional adverse events mentioned by both patients and HCPs but beyond the scope of the PRO-CTCAE were included in the recommendations section.

## Results

### Systematic literature review

**Figure 2:**
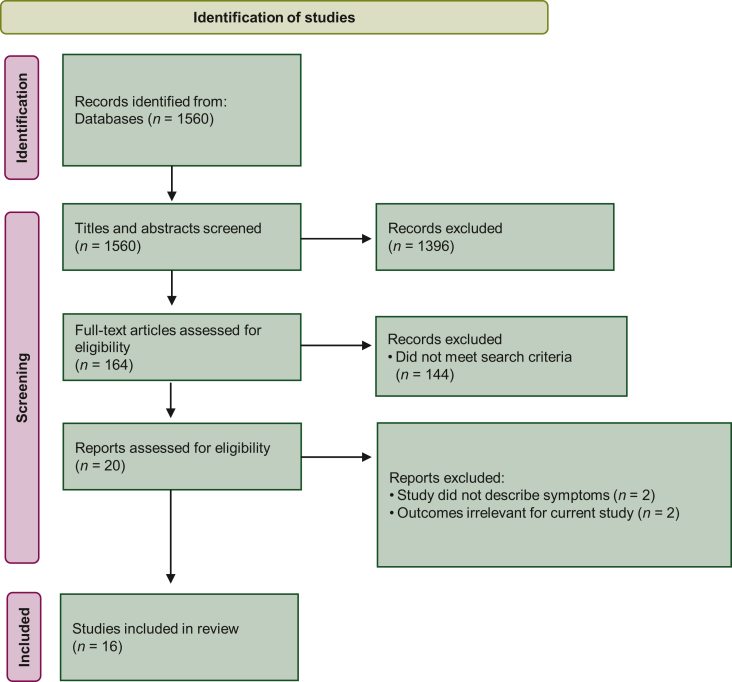
**Flowchart systematic literature review in PubMed.**

In the literature search, 1560 articles were identified. After screening titles and abstracts, 164 articles were included for full text screening. Twenty articles proved eligible; of these, 16 were considered relevant and included for data extraction (Figure 2, Supplementary Table S2 for reference list, available at https://doi.org/10.1016/j.esmoop.2022.100775). The included studies measured the following 21 adverse events that were available in the PRO-CTCAE item library: ‘urine incontinence’, ‘urinary urgency’, ‘urinary frequency’, ‘diarrhea’, ‘fecal incontinence’, ‘erectile dysfunction/impotence’, ‘hot flushes’, ‘constipation’, ‘bloating’, ‘flatulence’, ‘hair loss’, ‘nausea’, ‘skin irritation’, ‘reduced sexual desire’, ‘inability to reach orgasm’, ‘pain while urinating’, loss of libido’, ‘breast changes’, ‘fatigue’, ‘bowel problems’, ‘night sweats’, and ‘ejaculatory dysfunction’ (Supplementary Table S3, available at https://doi.org/10.1016/j.esmoop.2022.100775). Relevant adverse events beyond the scope of the PRO-CTCAE included ‘urinary retention’, ‘urinary tract pain’, ‘hematuria’, ‘fecal urgency’, ‘rectal hemorrhage’, ‘cystitis’, ‘bladder spasms’, ‘hemorrhoids’, ‘proctitis’, ‘abdominal cramps’, ‘mucus discharge’, ‘anal fissures’, ‘stress incontinence’, ‘back pain’, ‘urinary tract obstruction’, and ‘nocturia’ and were discussed for inclusion during the Delphi procedure.

### Semi-structured interviews: patients

Table 1 shows the characteristics of patients participating in the semi-structured interviews (*n* = 30). Their mean age was 71 years (range: 59-87 years). The majority of patients had completed higher vocational education (30%) or scientific education (57%). Patients were treated with a combination of hormone therapy with chemotherapy and/or radiotherapy (37%), hormone therapy (17%), radiotherapy only (17%), surgery (13%), and surgery with adjuvant radiotherapy (17%).

**Table 1:** Characteristics of patients participating in the semi-structured interviews (*n* = 30)

	*n* = 30 (%)
Age (years)	
Mean (SD)	71
Range	59-87
Type of care	
Curative	20 (66.6)
Palliative	10 (33.3)
Highest completed education	
Lower vocational education	2 (6.7)
High school	2 (6.6)
Higher vocational education	9 (30.0)
Scientific education	17 (56.7)
Marital status	
Married	20 (66.7)
Living together	6 (20.0)
Single	4 (13.3)
Treatment characteristics	
Combination therapy (Hormone therapy ± RT ± chemotherapy)	11 (36.7)
RT	5 (16.7)
Surgery	4 (13.3)
Surgery + RT	5 (16.7)
Hormone therapy	5 (16.7)
Treatment status	
On treatment	12 (40.0)
<1 month after treatment	5 (16.7)
1-3 months after treatment	4 (13.3)
3-6 months after treatment	6 (20.0)
>6 months after treatment	3 (10.0)

RT, radiotherapy; SD, standard deviation.

Patients were asked to score the relevance of each item on a four-point scale (1 = not relevant, 4 = highly relevant). The item considered most relevant was ‘Achieving and maintaining an erection’ (Mean: 2.83, standard deviation: 1.2). The following 11 items had a mean score ≥ 2: ‘urinary frequency’, ‘decreased libido’, ‘delayed orgasm’, ‘ejaculation’, ‘urinary urgency’, ‘unable to have orgasm’, ‘fatigue’, ‘urinary incontinence’, ‘flatulence’, ‘diarrhea’, and ‘painful urination’ (Supplementary Table S4, available at https://doi.org/10.1016/j.esmoop.2022.100775).

### Semi-structured interviews: HCP

Eight radiation oncologists, four urologists, two medical oncologists, and two nurse practitioners participated (*n* = 16). The included HCPs had varying work experience (ranging from 8 years to 33 years) and seven (43%) HCPs were female. From the HCPs’ perspective, ‘urinary incontinence’ was identified as most relevant (mean: 3.5)*.* Other relevant symptomatic adverse events, in order of the highest relevance, were: ‘urinary frequency’, ‘painful urination’, ‘ability to achieve and maintain erection’, ‘decreased libido’, ‘fatigue’, ‘urinary urgency’, ‘hot flashes’, ‘diarrhea’, ‘fecal incontinence’, ‘increased sweating’, and ‘ejaculation’. Items scored >2 by HCP but scored <2 by patients included ‘hot flashes’ and ‘fecal incontinence’, but also ‘insomnia’, ‘constipation’, ‘breast swelling and tenderness’, ‘general pain’, ‘stomach pain’, and ‘anxiety’ (Supplementary Table S4, available at https://doi.org/10.1016/j.esmoop.2022.100775).

### Delphi procedure

Fourteen HCPs (88%) and 12 patients (40%) agreed to participate in the first round. The results from all Delphi rounds are presented in Table 2. Overall consensus (≥75%) for inclusion was reached for five items: ‘ability to achieve and maintain erection’, ‘decreased libido’, ‘urinary frequency’, ‘urinary urgency’, and ‘urinary incontinence’. Items that were scored as relevant by HCP, but not by patients, were ‘fatigue’, ‘diarrhea’, ‘pain when urinating’, ‘hot flashes’, ‘fecal incontinence’, ‘breast swelling and tenderness’, and ‘feeling discouraged’. Items that were scored as relevant by patients, but not by the HCP, were ‘delayed orgasm’ and ‘ejaculation’. Twenty-six items were excluded. Consensus was not reached for 46 items, which were transferred to the second round.

**Table 2:** Item agreement in Delphi rounds for patients and health care providers

	Round 1, % yes			Round 2, % yes			Round 3, % yes		
	Pt	HCP	Total	Pt	HCP	Total	Pt	HCP	Total
Ability to achieve and maintain erection	100	92	96						
Urinary frequency	82	92	87						
Decreased libido	82	75	78						
Delayed orgasm	82	8	44		31	56	a		
Urinary urgency	73	92	83						
Ejaculation	91	33	61		62	77	a		
Inability to reach orgasm	55	58	57	67	46	56			
Fatigue	55	92	74	83		88			
Urinary incontinence	64	92	78						
Diarrhea	27	83	57	58		71			
Flatulence	46	50	48	25	39	32			
Painful urination	9	92	52	50		71			
Hot flashes	46	92	70	58		75			
Dry mouth	36	33	35	25	8	16			
Fecal incontinence	36	83	61	100		92			
Insomnia	18	42	30		15	16			
Feeling bloated	36	42	39	17	0	8			
Dizziness	46	25	35	25	15	20			
Change in urine color	9	17	13						
Increased sweating	36	25	30	17	0	8			
Numbness and tingling	27	41	35	17	23	20			
Dry skin	36	25	30	33	15	24			
Constipation	18	58	39		23	20			
Arthralgia	27	67	48	17	31	24			
Dyspnea	27	25	26	17	31	24			
Sadness	27	67	48	33	62	48			
Breast swelling and tenderness	18	75	48	33		54			
Voice changes	36	17	26	25		21			
Concentration	46	50	48	17	46	32			
Memory	36	50	44	8	46	28			
Decreased appetite	9	42	26		15	24			
Hoarseness	27	17	22						
General pain	0	33	17						
Muscle pain	27	50	39	8	23	16			
Stomach pain	18	58	39		23	20			
Discouraged	18	75	48	25		50			
Skin rash	18	33	26		15	16			
Cough	27	17	22						
Problems taste	18	25	22						
Wheezing	18	25	22						
Blurry vision	27	25	26	17	0	8			
Itching skin	46	25	35	25	8	16			
Anxiety	9	50	30	b	b	b			
Nausea	18	33	26						
Tinnitus	18	25	22						
Sensitivity to sunlight	27	25	26	25	8	16			
Aphthous ulcers	9	25	17						
Nail loss	9	25	17						
Heartburn	9	25	17						
Cold chills	9	42	26		15	12			
Bruises	27	33	30	25	15	20			
Headache	36	25	30	25	8	16			
Nose bleeds	9	25	17						
Darkening skin	9	25	17						
Visual floaters	55	17	35	33		25			
Swellings	9	42	26		15	12			
Pain swelling injection site	9	33	30						
Vomiting	18	42	22						
Palpitations	18	42	30		15	16			
Difficulty swallowing	9	25	17						
Skin reaction to irradiation	18	33	26		15	16			
Pimples on face or chest	9	25	17						
Change in nail color	18	25	22						
Chapped corners of mouth	27	25	26	25	8	16			
Hives	9	25	17						
Watery eyes	0	25	13						
Decreased sweating	0	17	9						
Hand foot syndrome	9	33	22						
Hiccups	0	17	9						
Ridged nails	0	25	13						
Hair loss	9	42	26		15	12			
Bedsores/pressure sores	9	17	13						
Light flashes	0	17	9						
Stretch marks/striae	18	25	22						
Body odor	9	17	13						

Numbers in each cell present the percentage of participants who scored an item as relevant.

If in round 1, an item received a consensus score of ≥75% from patients but not from HCPs, the item was only asked in the second round of the HCP survey, and vice versa; if an item scored between 25% and 75% in both expert groups, the item was asked in both HCPs and patients surveys.

Cells for round 3 are empty as no formal scoring took place (focus group meeting); Green: both ≥75% patients and HCP scored item as relevant, item was included; Yellow: either ≥75% of patients or HCP scored item as relevant, item was not (yet) included; Orange: both ≤ 25% of patients and HCP scored item as relevant, item was excluded; Gray: both 25%-75% patients and HCP scored item as relevant, item was not (yet) included.

HCP, health care provider; Pt, patients.

a These items were combined into the item ‘inability to reach orgasm’.

b Item was not displayed properly—no scores available in this round.

In the second round, 12/14 HCPs (86%) and 12/12 patients (100%) completed the survey. Overall consensus was reached to include four additional items: ‘ejaculation’, ‘fatigue’, ‘hot flashes’, and ‘fecal incontinence’. This led to nine included items in total. Five items were excluded, leading to 31 excluded items in total. Consensus was not reached for 11 items: ‘diarrhea’, ‘painful urination’, ‘delayed orgasm’, ‘inability to reach orgasm’, ‘breast swelling and tenderness’, ‘discouraged’, ‘sadness’, ‘flatulence’, ‘concentration’, ‘memory’, and ‘anxiety’*.*

The third Delphi round was a focus group expert meeting for which a heterogeneous group of experts involved in prostate cancer care (*N* = 10, radiation oncologists, urologists, medical oncologists, and nurse practitioners; and patient representatives, *n* = 2) participated. The experts agreed uniformly on the nine items included following round 2*.* From the 11 items for which no consensus was reached in the second round, the following six were included: ‘inability to reach orgasm’, ‘painful urination’, ‘feeling discouraged’, ‘sadness’, ‘anxiety’, and ‘concentration’*.* The following decisions were made. Firstly, ‘ejaculation’ was exchanged with ‘inability to reach orgasm’: Although ‘delayed orgasm’ and ‘inability to reach orgasm’ had the same relevance score (56%), the expert panel agreed that the spectrum of sexual dysfunction was best covered by the items ‘inability to reach orgasm’ and ‘ability to achieve and maintain erection’, making it redundant to add an extra item. Secondly, ‘painful urination’ was added due to its high score during the interviews and the first Delphi round. Furthermore, ‘feeling discouraged’ and ‘sadness’ were both added in order to cover the whole spectrum of patients’ emotional well-being; ‘anxiety’ was eliminated as it was covered by the former two items and scored lower than these. Lastly, low relevance was expressed for ‘concentration’ (32%) and ‘memory’ (28%) in the Delphi rounds, but the expert panel expressed that it was of clinical importance to include at least one item covering cognitive problems. ‘Concentration’ was favored over ‘memory’ based on the higher relevance score, correlation between the two items, and importance expressed by the patients. Eighty-two percent agreed on the 55 items that were excluded in the previous rounds; two participants (9%) did not answer this question.

Analyses stratified by treatment modality did not lead to the inclusion of additional items in the core subset. For radiotherapy, ‘bloating’, ‘sleeplessness’, ‘shortness of breath’, and ‘increased sweating’ were suggested to complement the subset. For radical prostatectomy, suggested items were ‘bloating’, ‘decreased appetite’, and ‘constipation’. For hormone therapy (with or without additional treatments), the suggested item was ‘dry mouth’. No items were suggested specifically for chemotherapy treatment.

### Finalization of the PRO-CTCAE subset

The final subset (Table 3) was presented by e-mail to the focus group meeting participants for formal agreement. The overall response rate was 70% [12/12 patients (100%) and 10/14 HCPs (71%)]. Ninety-one percent of participants agreed on the final subset, consisting of the following items: ‘ability to achieve and maintain erection’, ‘decreased libido’, ‘inability to reach orgasm’, ‘urinary frequency’, ‘urinary urgency’, ‘urinary incontinence’, ‘painful urination’, ‘fecal incontinence’, ‘fatigue’, ‘hot flashes’, ‘feeling discouraged’, ‘sadness’, and ‘concentration’. Lastly, several items had been identified in the literature, for which no PRO-CTCAE items were available. The experts agreed to add these (‘nocturia’, ‘blood and/or mucus in stool’, ‘hemorrhoids’, ‘hematuria’, ‘cystitis’, ‘neuropathy’, and ‘proctitis’) to the recommendation section of the core PRO-CTCAE subset*.*

**Table 3:** Final core outcome subset of PRO-CTCAE items and additionally recommended non-PRO-CTCAE items

Core outcome PRO-CTCAE items	Additionally recommended non-PRO-CTCAE items	Treatment-specific items
**Sexual functioning**		**Hormone therapy** a
• Ability to achieve and maintain erection		• Dry mouth
• Decreased libido		
• Inability to reach orgasm		
**Urination**		**Radiotherapy**
• Urinary frequency	• Nocturia	• Bloating
• Urinary urgency	• Hematuria	• Sleeplessness
• Urinary incontinence	• Cystitis	• Shortness of breath
• Painful urination		• Increased sweating
**Sleep/wake items**		**Radical prostatectomy**
• Fatigue		• Bloating
**Gastrointestinal items**		• Decreased appetite
• Fecal incontinence	• Proctitis	• Constipation
	• Hemorrhoids	
	• Blood and/or mucus in stool	
**Hormonal effects**		
• Hot flashes		
**Neurological**		
• Concentration	• Neuropathy	
**Mood**		
• Feeling discouraged		
• Sadness		

CTCAE, Common Terminology Criteria for Adverse Events; PRO, Patient-Reported Outcome.

a With or without additional treatment.

## Discussion

In this multicenter mixed-methods study, we developed a consensus-based PRO-CTCAE subset applicable for patient-reported adverse event monitoring during and after prostate cancer treatment. Out of 74 PRO-CTCAE items applicable to men, 13 items relevant in a multidisciplinary clinical setting for prostate cancer patients were included in the final outcome subset: ‘ability to achieve and maintain erection’, ‘decreased libido’, ‘inability to reach orgasm’, ‘urinary frequency’, ‘urinary urgency’, ‘urinary incontinence’, ‘painful urination’, ‘fecal incontinence’, ‘fatigue’, ‘hot flashes’, ‘feeling discouraged’, ‘sadness’, and ‘concentration’. The final subset was agreed on by 91% of the participants.

Assessment of adverse events and HRQoL in clinical practice yields interesting information, as it will give longitudinal data about the effects of treatment from an unselected population. Primary treatment decisions have a distinct impact on prostate cancer patients for a longer period after treatment.^6^ However, current evidence is still insufficient to precisely guide men about symptom risks and its subsequent effect on HRQoL.^20^ More specifically, the number of relevant studies is still limited, with a high heterogeneity in methodology, applied measurements, and outcome reporting.^6^^,^^20^ Measuring a pre-specified set of outcomes through a (selection of) validated outcome measure(s) following recommended methods of reporting could reduce study heterogeneity and lead to more specific treatment recommendations in the future.^20^

Toward this goal, several core outcome sets were developed already. On behalf of The Prostate Cancer Working Group from the NCI Symptom Management and Health-Related Quality-of-Life Steering Committee, Chen et al.^21^ suggested five items for clinical trials of localized prostate cancer patients (‘urinary incontinence’, ‘urinary obstruction and irritation’, ‘bowel-related symptoms’, ‘sexual dysfunction’, and ‘hormonal symptoms’) and four domains for advanced cancer (‘pain’, ‘fatigue’, ‘mental well-being’, and ‘physical well-being’). They, however, suggest a range of validated outcome measures to choose from, therefore still adding to the heterogeneity described above.^6^^,^^20^ The PRO-CTCAE subset developed in our study describes outcomes that are already operationalized in the PRO-CTCAE item library. Furthermore, Chen et al.^21^ describe that PRO data interpretation remains challenging, with the exact meaning of scores and score changes remaining unclear. A benefit of the PRO-CTCAE is the assessment of frequency, severity, and/or activity interference of each symptomatic adverse event^12^ that are combined into a CTCAE grade following a validated algorithm, where symptoms that worsened by ≥2 points or reached an absolute grade ≥3 are considered clinically relevant.^11^^,^^22^

The International Consortium for Health Outcomes Measurement developed two separate sets for prostate cancer including adverse events, HRQoL, baseline characteristics, and survival,^23^^,^^24^ but do not specifically state their rationale for this separation.^23^^,^^24^ Our subset is applicable to both localized and advanced prostate cancer as sensitivity analyses showed no notably different recommendations for which items to include.

We used a structured mixed-method approach based on the ‘phase 1 guideline for developing questionnaire modules’ by the EORTC Quality of Life group,^14^ allowing contributions from the literature, and patient and clinician perspectives. The current study may therefore serve as a guide for future development of tumor-specific PRO-CTCAE-based subsets incorporating the patients’ perspective in clinical monitoring of cancer treatments. Furthermore, our sample consisted of a clinical heterogeneous patient population across different time frames, reflecting the prostate cancer population. Even though patients treated with RT (with or without additional treatment modality) were slightly overrepresented, all other common treatment modalities were also represented. Therefore, the subset allows supervision of patient adverse events during the full course of treatment and the subsequent follow-up trajectory.

The present study is limited by the national setting; international multicenter verification is still required in order to test psychometric properties. Nonetheless, since international treatment guidelines are largely comparable and the literature review revealed no additional adverse events, the subset will probably be useful in an international setting as well. Furthermore, a relatively large proportion of highly educated patients was included. As health literacy is correlated with education level, but can also differ among age and sex,^25^^,^^26^ our study would have benefitted from including patients with a variety of educational levels. Nonetheless, the PRO-CTCAE questionnaire is validated in a heterogeneous patient group with varying educational levels. Therefore, the subset will probably be applicable across all educational levels. Educational levels may have affected the patients’ performance status before their cancer diagnosis, and the prevalence of comorbid diseases. This may have affected the adverse effects patients and HRQoL patients experienced and thus reported, with higher educated people reporting more favorable HRQoL,^27^ leading to an underestimation of the adverse effects included in the subset. We have partially evaded this by including patients in a palliative care setting, but interpretation of results could be improved by information on the patients’ baseline health status.

### Conclusions

To conclude, this study presents a PRO-CTCAE-based subset for multidisciplinary adverse events monitoring in prostate cancer patients during and after cancer treatment. It is anticipated that monitoring adverse events with the current PRO-CTCAE based subset for prostate cancer patients will ultimately provide a more representative reflection of patients’ experiences. Furthermore, the current study may serve as a guide for future development of tumor-specific PRO-CTCAE-based subsets incorporating the patients’ perspective in clinical monitoring of cancer treatments.

The use of the PRO-CTCAE subset could facilitate remote monitoring: the current study was carried out during the COVID-19 pandemic, which widely impacted the health care system. Clinical implementation of the PRO-CTCAE subset could enable remote monitoring while providing crucial patient care and decreasing the risk of spreading the virus during direct contact between patient and HCPs. Furthermore, considering the high prevalence of prostate cancer,^2^^,^^4^^,^^28^ remote monitoring could also take off pressure of prostate cancer care in general. This highlights the importance of the use of PRO tools for future clinical care.

## Glossary

• Common Terminology Criteria for Adverse Events (CTCAE): reporting system for adverse events reporting, completed by clinicians.
• Delphi procedure: method aimed at reaching consensus in a structured way, based on experts’ opinions on a specific subject. Information and feedback is gathered through multiple questionnaire rounds, which are completed anonymously to avoid bias that may result from desire to group conformity. The method is targeted at making best use of already available information.
• Patient-Reported Outcomes (PROs): Validated questionnaires about health-related quality of life (e.g. functioning, symptoms, adverse events, etc.) that are completed by patients.
