# Supplementary material for: Selecting a PRO-CTCAE-based subset for patient-reported symptom monitoring in prostate cancer patients: a modified Delphi procedure
Source: ESMO Open. 2023 Jan 16;8(1):100775. doi: 10.1016/j.esmoop.2022.100775 (PMC10024147; doi:10.1016/j.esmoop.2022.100775)
Supplement: Supplementary Table [file mmc1.docx]

**Appendices**

**Supplementary Table 1: full search strategy for comprehensive literature review**

| #1 AND #2 AND #3,,Full text,"(""Prostatic Neoplasms""[MeSH Major Topic] OR ((""prostatic""[Title] OR ""prostate""[Title]) AND (""neoplasm*""[Title] OR ""tumor""[Title] OR ""tumors""[Title] OR ""tumour*""[Title] OR ""cancer*""[Title] OR ""malignan*""[Title] OR ""oncolog*""[Title] OR ""carcinom*""[Title]))) AND (""Drug-Related Side Effects and Adverse Reactions""[MeSH Major Topic] OR ""Drug Eruptions""[MeSH Major Topic] OR ""Toxic Actions""[MeSH Major Topic] OR ""dose response relationship, drug""[MeSH Major Topic] OR ""Treatment Failure""[MeSH Major Topic] OR ""Contraindications""[MeSH Major Topic] OR ""complication*""[Title] OR ""adverse effect*""[Title] OR ""adversely affect*""[Title] OR ""side effect*""[Title] OR ""contraindicat*""[Title] OR ""adverse drug reaction*""[Title] OR ""drug eruption*""[Title] OR ""toxic effect*""[Title] OR ""adverse event*""[Title] OR ""toxic*""[Title]) AND (""Radiotherapy""[MeSH Terms] OR ""radiotherap*""[Title/Abstract] OR ""radiation""[Title/Abstract] OR ""irradiat*""[Title/Abstract] OR ""gammaknife""[Title/Abstract] OR ""cyberknife""[Title/Abstract] OR ""stereotactic""[Title/Abstract] OR ""SRS""[Title/Abstract] OR ""SRT""[Title/Abstract] OR ""SBRT""[Title/Abstract] OR ""radiosurger*""[Title/Abstract] OR ""chemoradi*""[Title/Abstract] OR ""radiochemo*""[Title/Abstract] OR ""radioimmuno*""[Title/Abstract] OR ""surgical procedures, operative""[MeSH Terms] OR ""surger*""[Title/Abstract] OR ""surgical""[Title/Abstract] OR ""operation*""[Title/Abstract] OR ""operative""[Title/Abstract] OR ""prostatectom*""[Title/Abstract] OR ""resection""[Title/Abstract] OR ""chemoradiotherapy""[MeSH Terms] OR ""chemotherapy, adjuvant""[MeSH Terms] OR ""adjuvant chemotherap*""[Title/Abstract] OR ""adjuvant drug therap*""[Title/Abstract] OR ""chemoradiotherap*""[Title/Abstract] OR ""radiochemotherap*""[Title/Abstract] OR ""chemoradiation""[Title/Abstract] OR ""chemotherap*""[Title/Abstract] OR ""chemo-radiation""[Title/Abstract] OR ""Hormone Replacement Therapy""[MeSH Terms] OR ""hormonal therap*""[Title/Abstract] OR ""hormone therap*""[Title/Abstract] OR ""hormone replacement therap*""[Title/Abstract])","1,560",07:48:51 |
| --- |
| #3,"""Radiotherapy""[Mesh] OR radiotherap*[tiab] OR radiation[tiab] OR irradiat*[tiab] OR gammaknife[tiab] OR cyberknife[tiab] OR stereotactic[tiab] OR SRS[tiab] OR SRT[tiab] OR SBRT[tiab] OR radiosurger*[tiab] OR chemoradi*[tiab] OR radiochemo*[tiab] OR radioimmuno*[tiab] OR ""Surgical Procedures, Operative""[Mesh] OR surger*[tiab] OR surgical[tiab] OR operation*[tiab] OR operative[tiab] OR prostatectom*[tiab] OR resection[tiab] OR Chemoradiotherapy[Mesh] OR ""Chemotherapy, Adjuvant""[Mesh] OR Adjuvant Chemotherap* [tiab] OR Adjuvant Drug Therap* [tiab] OR chemoradiotherap* [tiab] OR radiochemotherap* [tiab] OR chemoradiation [tiab] OR chemotherap* [tiab] OR chemo-radiation [tiab] OR ""Hormone Replacement Therapy""[Mesh] OR hormonal therap*[tiab] OR hormone therap*[tiab] OR hormone replacement therap*[tiab]",,,"""Radiotherapy""[MeSH Terms] OR ""radiotherap*""[Title/Abstract] OR ""radiation""[Title/Abstract] OR ""irradiat*""[Title/Abstract] OR ""gammaknife""[Title/Abstract] OR ""cyberknife""[Title/Abstract] OR ""stereotactic""[Title/Abstract] OR ""SRS""[Title/Abstract] OR ""SRT""[Title/Abstract] OR ""SBRT""[Title/Abstract] OR ""radiosurger*""[Title/Abstract] OR ""chemoradi*""[Title/Abstract] OR ""radiochemo*""[Title/Abstract] OR ""radioimmuno*""[Title/Abstract] OR ""surgical procedures, operative""[MeSH Terms] OR ""surger*""[Title/Abstract] OR ""surgical""[Title/Abstract] OR ""operation*""[Title/Abstract] OR ""operative""[Title/Abstract] OR ""prostatectom*""[Title/Abstract] OR ""resection""[Title/Abstract] OR ""chemoradiotherapy""[MeSH Terms] OR ""chemotherapy, adjuvant""[MeSH Terms] OR ""adjuvant chemotherap*""[Title/Abstract] OR ""adjuvant drug therap*""[Title/Abstract] OR ""chemoradiotherap*""[Title/Abstract] OR ""radiochemotherap*""[Title/Abstract] OR ""chemoradiation""[Title/Abstract] OR ""chemotherap*""[Title/Abstract] OR ""chemo-radiation""[Title/Abstract] OR ""Hormone Replacement Therapy""[MeSH Terms] OR ""hormonal therap*""[Title/Abstract] OR ""hormone therap*""[Title/Abstract] OR ""hormone replacement therap*""[Title/Abstract]","5,293,587",07:40:53 |
| #2,"""Drug-Related Side Effects and Adverse Reactions""[Majr] OR ""Drug Eruptions""[Majr] OR ""Toxic Actions""[Majr] OR ""Dose-Response Relationship, Drug""[Majr] OR ""Treatment Failure""[Majr] OR ""Contraindications""[Majr] OR complication*[ti] OR adverse effect*[ti] OR adversely affect*[ti] OR side effect*[ti] OR contraindicat*[ti] OR adverse drug reaction*[ti] OR drug eruption*[ti] OR toxic effect*[ti] OR adverse event*[ti] OR toxic*[ti]",,,"""Drug-Related Side Effects and Adverse Reactions""[MeSH Major Topic] OR ""Drug Eruptions""[MeSH Major Topic] OR ""Toxic Actions""[MeSH Major Topic] OR ""dose response relationship, drug""[MeSH Major Topic] OR ""Treatment Failure""[MeSH Major Topic] OR ""Contraindications""[MeSH Major Topic] OR ""complication*""[Title] OR ""adverse effect*""[Title] OR ""adversely affect*""[Title] OR ""side effect*""[Title] OR ""contraindicat*""[Title] OR ""adverse drug reaction*""[Title] OR ""drug eruption*""[Title] OR ""toxic effect*""[Title] OR ""adverse event*""[Title] OR ""toxic*""[Title]","782,631",07:39:53 |
| #1,"""Prostatic Neoplasms""[Majr] OR ((prostatic[ti] OR prostate[ti]) AND (neoplasm* [ti] OR tumor [ti] OR tumors [ti] OR tumour* [ti] OR cancer* [ti] OR malignan* [ti] OR oncolog* [ti] OR carcinom*[ti]))",,,"""Prostatic Neoplasms""[MeSH Major Topic] OR ((""prostatic""[Title] OR ""prostate""[Title]) AND (""neoplasm*""[Title] OR ""tumor""[Title] OR ""tumors""[Title] OR ""tumour*""[Title] OR ""cancer*""[Title] OR ""malignan*""[Title] OR ""oncolog*""[Title] OR ""carcinom*""[Title]))","128,354",07:39:37 |

**Supplementary Table 2: reference list systematic literature review**

| **Authors** | **Year** | **Title** | **Reference** |
| --- | --- | --- | --- |
| Boyer, J., et al. | 2017 | Toxicity and quality of life report of a phase II study of stereotactic body radiotherapy (SBRT) for low and intermediate risk prostate cancer | Radiat Oncol. 2017 Jan 13;12(1):14. doi: 10.1186/s13014-016-0758-8. |
| Chin S., et al. | 2017 | Toxicity after post-prostatectomy image-guided intensity-modulated radiotherapy using Australian guidelines | Journal of Medical Imaging and Radiation Oncology 61 (2017) 804–811 |
| Dearnaley D., et al. | 2018 | Toxicity and Patient-Reported Outcomes of a  Phase 2 Randomized Trial of Prostate and Pelvic  Lymph Node Versus Prostate only Radiotherapy  in Advanced Localised Prostate Cancer (PIVOTAL) | Int J Radiation Oncol Biol Phys, Vol. 103, No. 3, pp. 605e617, 2019 |
| Devlin, E. J. et al. | 2019 | ‘‘Just As I Expected’’: A Longitudinal Cohort Study of the Impact of Response Expectancies on Side Effect Experiences During Radiotherapy for Prostate Cancer | Journal of Pain and Symptom Management, Volume 57, Issue 2, February 2019, Pages 273-281.e4 |
| Dutz A., et al. | 2019 | Early and late side effects, dosimetric parameters and quality of life after proton beam therapy and IMRT for prostate cancer: a matched-pair analysis | Acta Oncol. 2019 Jun;58(6):916-925. doi: 10.1080/0284186X.2019.1581373. Epub 2019 Mar 18. |
| Heemsbergen W., et al. | 2020 | Local Dose Effects for Late Gastrointestinal Toxicity After Hypofractionated and Conventionally Fractionated Modern Radiotherapy for Prostate Cancer in the HYPRO Trial | Front. Oncol. 10:469.  doi: 10.3389/fonc.2020.00469 |
| Hunter K., et al. | 2012 | Long-term (10-year) gastrointestinal and genitourinary toxicity after treatment with external beam radiotherapy, radical prostatectomy, or brachytherapy for prostate cancer | Prostate Cancer. 2012;2012:853487. doi: 10.1155/2012/853487. Epub 2012 Apr 11. |
| Kozuka T., et al. | 2017 | Acute and late complications after hypofractionated intensity modulated radiotherapy in prostate cancer | Jpn J Radiol (2017) 35:269–278. DOI 10.1007/s11604-017-0630-2 |
| Mohammed, W., et al. | 2018 | Short-term Toxicity of High Dose Rate Brachytherapy in Prostate Cancer Patients with Inflammatory Bowel Disease | Clinical Oncology 30 (2018) 534e538 |
| Sakurai T., et al. | 2020 | Toxicity and clinical outcomes of single-fraction high-dose-rate brachytherapy combined with external beam radiotherapy for high-/very high-risk prostate cancer: A dosimetric analysis of toxicity | Jpn J Radiol. 2020 Dec;38(12):1197-1208. doi: 10.1007/s11604-020-01023-2. Epub 2020 Jul 31. |
| Savard J., et al. | 2013 | Prostate cancer treatments and their side effects are associated with increased insomnia | Psychooncology. 2013 Jun;22(6):1381-8. doi: 10.1002/pon.3150. |
| Schlussel Markovic E., et al. | 2018 | Outcomes and toxicities in patients with intermediate-risk prostate cancer treated with brachytherapy alone or brachytherapy and supplemental external beam radiation therapy | BJU Int. 2018 May;121(5):774-780. doi: 10.1111/bju.14128. Epub 2018 Feb 16. |
| Schorghofer, A., et al. | 2019 | Risk-adapted moderate hypofractionation of prostate cancer: A prospective analysis of acute toxicity, QOL and outcome in 221 patients | Strahlenther Onkol (2019) 195:894–901 |
| Steentjes, L., et al. | 2018 | Factors associated with current and severe physical side-effects after prostate cancer treatment: What men report | Eur J Cancer Care (Engl). 2018 Jan;27(1). doi: 10.1111/ecc.12589. |
| Vaugier L.. et al. | 2019 | Early Toxicity of a Phase 2 Trial of Combined Salvage Radiation Therapy and Hormone Therapy in Oligometastatic Pelvic Node Relapses of Prostate Cancer (OLIGOPELVIS GETUG P07) | Int J Radiat Oncol Biol Phys  . 2019 Apr 1;103(5):1061-1067. |
| Vogel M. E., et al. | 2019 | Adjuvant versus early salvage radiotherapy: outcome of patients with prostate cancer treated with postoperative radiotherapy after radical prostatectomy | Radiat Oncol . 2019 Nov 11;14(1):198. doi: 10.1186/s13014-019-1391-0. |

Supplementary Table 3. Overview of included studies from the systematic literature review (n=16) and adverse events extracted from these studies

| **Authors** | **Year** | **Design** | **Treatment** | **Sample size** | **Patient population** | **Survey** | **Adverse events** |
| --- | --- | --- | --- | --- | --- | --- | --- |
| Boyer, J., et al. | 2017 | Phase II clinical trial | RT (low vs. intermediate risk prostate cancer) | 60 | 60 patients with T1–T2c prostate cancer with a Gleason score of 6 and PSA ≤ 15 or Gleason score of 7 and PSA ≤ 10 | Symptoms: CTCAE v4.0;  HRQOL: American Urological Association symptom score, IIEF, EPIC-SF | Urinary frequency, urinary urgency, urine incontinence. *Urinary retention, urinary tract pain, hematuria, fecal urgency, rectal hemorrhage*. (patient-reported) |
| Chin S., et al. | 2017 | Retrospective cohort study | Prostatectomy with adjuvant RT and hormone therapy (ADT) | 293 | Men who underwent 64–66 Gy IG-IMRT to the prostate bed between 2007 and 2015; Patients were excluded if they had macroscopic recurrence, pathologically or clinically involved pelvic lymph nodes or metastatic disease. | Symptoms: CTCAE v4.0; | Urinary frequency, urinary incontinence, urinary urgency, diarrhea, fecal incontinence, ejaculatory dysfunction, erectile dysfunction, orgasm dysfunction. *Cystitis, bladder spasms,* *urinary retention,* *hemorrhoids, proctitis. (clinician-reported)* |
| Dearnaley D., et al. | 2018 | Phase II randomized clinical trial | Conventional RT vs. conenventional RT on the prostate and a lower dose on pelvic lymph nodes | 124 | Patients with locally advanced, high-risk prostate cancer (localized adenocarcinoma of the prostate, stage T3b/T4) | Symptoms: CTCAE v4.0;  HRQoL: IBDQ, Vaizey Incontinence  Questionnaire,IPSS | Diarrhea, urine incontinence, urinary urgency, and urinary frequency. *Proctitis, rectal bleeding, cystitis*, *urine retention, inability to pass urine. (clinician-reported)* |
| Devlin, E. J. et al. | 2019 | Prospective cohort study | Conventional RT (74 to 78 Gy) | 35 | Male outpatients diagnosed with Stage I-III prostate cancer | HRQoL: DASS21, Mental Adjustment to Cancer scale | Urinary urgency, urinary frequency, urine incontinence, hair loss, fatigue, nausea, skin irritation, decreased libido, inability to reach orgasm, inability to have or maintain erection. *Bowel leakage, abdominal cramps,* *rectal urgency, painful bowel movement, blood in stools. (patient-reported)* |
| Dutz A., et al. | 2019 | Prospective cohort study | Proton beam therapy vs. RT | 88 | Histologically confirmed localized or locally advanced prostate cancer without positive pelvic lymph nodes or distant metastases | Symptoms: CTCAE v4.0;  HRQoL: EORTC-QLQ-C30/PR25 | Pain, urinary frequency, urinary urgency, urinary incontinence, diarrhea, fecal incontinence. *Cystitis, hematuria, obstruction, proctitis, rectal bleeding* (clinician-reported) |
| Heemsbergen W., et al. | 2020 | Phase III randomized clinical trial | Hypofractionated RT (78Gy) vs. conventional RT (64Gy) | 633 | T1-4 | Not specified | Fecal incontinence. *Rectal bleeding,* *increased stool frequency, mucus discharge,* *pain with stools. (patient-reported)* |
| Hunter K., et al. | 2012 | Retrospective cohort study | Radical prostatectomy, brachytherapy or RT | 525 | Patients treated in 1999; 24% patients treated with prostate interstitial brachytherapy, 40% with radical prostatectomy, and 36% with external beam radiation therapy. | Symptoms: CTCAE v4.0; | Urinary incontinence, fecal incontinence. *Hematuria, rectal bleeding (clinician-reported)* |
| Kozuka T., et al. | 2017 | Prospective cohort study | Hypofractionated RT (70 Gy) vs. conventional RT (78Gy) | 117 | Patients with intermediate-risk localized prostate cancer, histologically confirmed T1-T2N0M0 | Symptoms: CTCAE v4.0;  HRQoL: IPSS | Urinary frequency, urinary urgency. *Anal pain,* *urinary retention,* *anal fissures, rectal hemorrhage (clinician-reported)* |
| Mohammed, W., et al. | 2018 | Prospective cohort study | High-dose brachytherapy | 11 | T1beT3b disease and any Gleason score | Symptoms: CTCAE v4.0; | Diarrhea, urinary frequency, urinary urgency.  *Proctitis, cystitis, urinary retention. (clinician-reported)* |
| Sakurai T., et al. | 2020 | Retrospective cohort study | Brachytherapy with adjuvant RT (46 Gy), neoadjuvant hormone therapy (plus adjuvant hormone therapy in high-risk patients) | 124 | Patients with high or very high-risk disease; Patients with lymph node or distant metastases (N1 or M1) were excluded. | Symptoms: CTCAE v4.0;  HRQoL: IPSS, 7-grade Quality of Life Scale | Urinary frequency, urinary urgency, urinary incontinence, diarrhea. *Rectal hemorrhage, hematuria,* *urinary retention, urinary tract pain. (clinician-reported)* |
| Savard J., et al. | 2013 | Prospective cohort study | Hormone therapy (ADT) with RT vs RT only | 60 | Men scheduled to receive RTH for prostate cancer, with (n = 28) or without (n = 32) ADT, stage I, II, and III | Insomnia Severity Index and the Physical Symptoms Questionnaire | Hot flashes, urinary frequency, insomnia, night sweats. (patient-reported) |
| Schlussel Markovic E., et al. | 2018 | Prospective cohort study | Brachytherapy ± RT ± hormone therapy (ADT) | 902 | PSA 10–20 ng/mL; Gleason score 7; or stage T2b or T2c | IPSS, SHIM, RTOG rectal bleeding scale, and Mount Sinai erectile function scale, | Urinary incontinence. *Dysuria, hematuria, stress incontinence, rectal bleeding, urinary retention.*   (clinician-reported) |
| Schorghofer, A., et al. | 2019 | Prospective cohort study | Risk-adapted hypofractioned RT | 221 | Hypofractionated treatment patients had to have an IPSS score <12 | Symptoms: CTCAE v4.0;  HRQoL: IPSS, EORTC-QLQ-PR25 | Urinary frequency, urinary urgency, urinary incontinence, diarrhea, fecal incontinence. *Hematuria,* *urinary retention, proctitis, rectal hemorrhage and rectal ulcer. (clinician-reported)* |
| Steentjes, L., et al. | 2018 | Cross-sectional survey study in prostate cancer survivors | Prostatectomy, RT, hormone therapy, brachytherapy, active surveillance/watchful-waiting | 3348 | All stages. | Not specified | Urinary frequency, pain while urinating, erectile dysfunction, decreased libido, impotence, urinary incontinence, breast changes, hot flushes, fatigue, bowel problems. *Blood in urine,* *back pain. (patient-reported)* |
| Vaugier L., et al. | 2019 | Phase II clinical trial | Radical prostatectomy with adjuvant RT | 74 | PT1-3, PN0-1 | Symptoms: CTCAE v4.0;  HRQoL: EORTC QLQ-C30 and QLQ-PR25 | Urinary urgency, urinary incontinence, diarrhea, constipation, bloating and flatulence. *Abdominal pain*, *rectal bleeding and hematuria. (clinician-reported)* |
| Vogel M. E., et al. | 2019 | Retrospective cohort study | Radical prostatectomy with adjuvant or salvage RT | 253 | Patients treated with adjuvant radiotherapy; PT2a-4, PN0-1 | Not specified | Diarrhea, fecal incontinence, urinary incontinence, erectile dysfunction. *Proctitis,* *rectal bleeding, cystitis, hematuria, urinary tract obstruction, nocturia. (clinician-reported)* |
| Items in *italics* were extracted from literature, but are (currently) not represented in the PRO-CTCAE item library  Abbreviations:   - PPRT: post-prostatectomy radiotherapy - RT: radiotherapy - ADT: androgen deprivation therapy - IG-IMRT: image-guided intensity-modulated radiation therapy - CTCAE: Common Terminology Criteria for Adverse Events - HRQoL: Health-related quality of life - IIEF: International Index of Erectile Function - EPIC-SF: Expanded Prostate cancer Index Composite Short Form - IBDQ: Inflammatory Bowel Disease Questionnaire - IPSS: International Prostate Symptom Score - DASS21: Depression, Anxiety and Stress Scale 21 - EORTC-QLQ-C30/PR25: European Organisation for Research and Treatment of Cancer - SHIM: Sexual Health Inventory for Men | | | | | | | |

Supplementary Table 4: Relevance scores for each PRO-CTCAE item, assessed by patients (n=30) and HCP (n=16).

| PRO-CTCAE item | Mean relevance score (range: 0-4) | |
| --- | --- | --- |
|  | Patients (n=30) | HCP (n=16) |
| Ability to achieve and maintain erection | 2.82 | 3.31 |
| Urinary frequency | 2.71 | 3.38 |
| Decreased libido | 2.68 | 3.25 |
| Delayed orgasm | 2.5 | 2.13 |
| Urinary urgency | 2.46 | 3.25 |
| Ejaculation | 2.46 | 2.63 |
| Inability to reach orgasm | 2.43 | 2.56 |
| Fatigue | 2.29 | 3.25 |
| Urinary incontinence | 2.18 | 3.5 |
| Diarrhea | 2.11 | 2.94 |
| Flatulence | 2.11 | 1.88 |
| Painful urination | 2.04 | 3.31 |
| Hot flashes | 1.86 | 3.13 |
| Dry mouth | 1.82 | 1.56 |
| Fecal incontinence | 1.68 | 2.88 |
| Insomnia | 1.68 | 2.13 |
| Feeling bloated | 1.68 | 1.88 |
| Dizziness | 1.64 | 1.88 |
| Change in urine color | 1.61 | 1.75 |
| Increased sweating | 1.57 | 2.75 |
| Numbness and tingling | 1.57 | 2.19 |
| Dry skin | 1.57 | 1.56 |
| Constipation | 1.54 | 2.56 |
| Arthralgia (joint pain) | 1.54 | 2.19 |
| Dyspnoea (shortness of breath) | 1.54 | 1.94 |
| Sadness | 1.5 | 2.56 |
| Breast swelling and tenderness | 1.5 | 2.44 |
| Voice changes | 1.5 | 1.44 |
| Concentration | 1.43 | 2.31 |
| Memory | 1.43 | 2.19 |
| Decreased appetite | 1.43 | 1.81 |
| Hoarseness | 1.43 | 1.25 |
| General pain | 1.39 | 2.56 |
| Muscle pain | 1.39 | 1.88 |
| Stomach pain | 1.36 | 2.5 |
| Discouraged | 1.36 | 2.0 |
| Skin rash | 1.36 | 1.63 |
| Cough | 1.36 | 1.63 |
| Problems taste | 1.32 | 1.75 |
| Wheezing | 1.32 | 1.69 |
| Blurry vision | 1.32 | 1.69 |
| Itching skin | 1.32 | 1.63 |
| Anxiety | 1.29 | 2.44 |
| Nausea | 1.29 | 2 |
| Tinnitus | 1.29 | 1.69 |
| Sensitivity to sunlight | 1.29 | 1.63 |
| Aphthous ulcers | 1.25 | 1.69 |
| Nail loss | 1.25 | 1.69 |
| Heartburn | 1.25 | 1.5 |
| Cold chills | 1.21 | 2.19 |
| Bruises | 1.21 | 1.75 |
| Headache | 1.21 | 1.5 |
| Nose bleeds | 1.21 | 1.5 |
| Darkening skin | 1.21 | 1.38 |
| Visual floaters | 1.21 | 1.13 |
| Swellings | 1.18 | 2.31 |
| Pain swelling injection site | 1.18 | 2 |
| Vomiting | 1.18 | 1.81 |
| Palpitations | 1.18 | 1.81 |
| Difficulty swallowing | 1.18 | 1.56 |
| Skin reaction to irradiation | 1.18 | 1.56 |
| Pimples on face or chest | 1.18 | 1.38 |
| Change in nail color | 1.18 | 1.38 |
| Chapped corners of mouth | 1.18 | 1.31 |
| Hives | 1.18 | 1.31 |
| Watery eyes | 1.18 | 1.25 |
| Decreased sweating | 1.18 | 1.19 |
| Hand foot syndrome | 1.14 | 1.63 |
| Hiccups | 1.14 | 1.38 |
| Ridged nails | 1.14 | 1.25 |
| Hair loss | 1.11 | 2.13 |
| Bedsores/pressure sores | 1.11 | 1.69 |
| Light flashes | 1.11 | 1.44 |
| Body odour | 1.07 | 1.44 |
| Stretch marks/striae | 1.07 | 1.13 |
| Ordered from highest to lowest scores for patients, mean relevance scores ≥2 highlighted in green.  Abbreviations: HCP=health care provider | | |
